# Prevalence and types of inconsistencies in clinical pharmacogenetic recommendations among major U.S. sources

**DOI:** 10.1038/s41525-020-00156-7

**Published:** 2020-10-30

**Authors:** Tyler Shugg, Amy L. Pasternak, Bianca London, Jasmine A. Luzum

**Affiliations:** 1 Department of Clinical Pharmacy, University of Michigan College of Pharmacy, Ann Arbor, MI USA; 2 Division of Clinical Pharmacology, Indiana University School of Medicine, Indianapolis, IN USA; 3 Senior Health Services at Blue Cross Blue Shield of Michigan Emerging Markets, Southfield, MI USA

**Keywords:** Pharmacogenetics, Molecularly targeted therapy

## Abstract

Clinical implementation of pharmacogenomics (PGx) is slow. Previous studies have identified some inconsistencies among clinical PGx recommendations, but the prevalence and types of inconsistencies have not been comprehensively analyzed among major PGx guidance sources in the U.S. PGx recommendations from the Clinical Pharmacogenetics Implementation Consortium, U.S. Food and Drug Administration drug labels, and major U.S. professional medical organizations were analyzed through May 24, 2019. Inconsistencies were analyzed within the following elements: recommendation category; whether routine screening was recommended; and the specific biomarkers, variants, and patient groups involved. We identified 606 total clinical PGx recommendations, which contained 267 unique drugs. Composite inconsistencies occurred in 48.1% of clinical PGx recommendations overall, and in 93.3% of recommendations from three sources. Inconsistencies occurred in the recommendation category (29.8%), the patient group (35.4%), and routine screening (15.2%). In conclusion, almost one-half of clinical PGx recommendations from prominent U.S. guidance sources contain inconsistencies, which can potentially slow clinical implementation.

## Introduction

Advances in pharmacogenetics (PGx) over the past two decades have rapidly enhanced our understanding of the effects of genetics on drug disposition, therapeutic efficacy, and toxicity. However, the clinical potential of PGx has remained largely unrealized. Clinical implementation of PGx has been primarily confined to large academic medical centers^1,2^, and wider implementation has been slow and non-routine. Research from academic implementation efforts has shown that the major factors slowing the widespread implementation of PGx include lack of clinician PGx training and lack of clinician confidence in interpreting PGx results^3–6^. These findings are consistent with the results from a nationwide survey in 2012 that found ~90% of U.S. physicians felt inadequately informed about PGx testing^7^.

In order to overcome these barriers related to deficits in clinician PGx training and knowledge, clinical PGx recommendations from major U.S. guidance sources need to be clear and consistent^8^. When the physician survey was published in 2012, few clinical PGx recommendations had been published at the time. Since then, hundreds of clinical PGx recommendations have been published from a variety of sources. Currently in the U.S., clinicians primarily receive PGx clinical recommendations from three major sources: guidelines published by the Clinical Pharmacogenetics Implementation Consortium (CPIC); U.S. Food and Drug Administration (FDA) drug labels; and clinical practice guidelines (CPGs) published by professional medical organizations and technology assessors (e.g., the National Comprehensive Cancer Network (NCCN) and Evaluation of Genomic Applications in Practice and Prevention Working Group (EGAPP)). Previous studies have identified some inconsistencies among published clinical PGx recommendations^8–10^. Inconsistencies among clinical PGx recommendations make it challenging for the clinician to decide which recommendations to follow. To our knowledge, the prevalence and types of inconsistencies among current clinical PGx recommendations from the major U.S. sources (CPIC, FDA, and CPGs) have not been thoroughly investigated. This issue is perhaps more timely than ever since the FDA recently approved direct-to-consumer PGx testing by the company 23andMe^11^. While FDA guidance warns that preliminary findings from 23andMe should be confirmed with clinical genetic testing in order to inform medical decision-making, the FDA approval does identify a role for 23andMe PGx reports to “enable users to access information about their genetics that could aid discussions with a healthcare professional.”^11^ Over 10 million individuals have been genotyped by 23andMe. Moreover, several healthcare systems have begun implementing biobanks, in which genomic data can be directly linked to electronic medical records^12^. Therefore, healthcare providers need clear recommendations for how to use PGx test results. The objective of this study was to characterize the prevalence and types of inconsistencies among current clinical PGx recommendations from the three major U.S. sources: CPIC, FDA, and CPGs.

## Results

### Characteristics of PGx recommendations overall and compared by source

A total of 606 current PGx recommendations were identified and categorized (see Supplementary Information for the complete dataset). Table 1 describes the recommendations overall and in the following strata: germline vs. somatic vs. pathogen; pharmacokinetic (PK) vs. pharmacodynamic (PD); genetic variant vs. gene/protein expression; and therapeutic area (e.g., oncology, cardiology, rheumatology). Overall, most PGx recommendations were based on germline (58.3%), PD (68.7%), and genetic variants (79.9%). The therapeutic area that had the most PGx recommendations was oncology (35.8%), and the most common recommendation category was Indication (32.0%). Sixty-seven percent of PGx recommendations overall were categorized as “actionable”, and 34.0% recommended routine screening. (Data on routine screening does not include CPIC because CPIC does not recommend whether or not to order a PGx test). The median (interquartile range (IQR)) time since the publication of the PGx recommendations overall was 0.75 (0.19–1.77) years, and the median (IQR) number of variants specified in the PGx recommendations overall was 0 (0–2).

**Table 1:** Characteristics of all PGx recommendations and stratified by source of the recommendation.

Strata	All PGx Recs (*n* = 606)	Source of PGx Recs			*P* value*
		CPG (*n* = 172)	CPIC (*n* = 66)	FDA (*n* = 368)	
# unique drugs	267	91	46	254	n/a
# unique drug–gene pairs	433	172	66	368	n/a
*Germline vs. somatic vs. pathogen*					
Germline	353 (58.3%)	50 (29.1%)	66 (100.0%)	237 (64.4%)	**<0.001**
Somatic	250 (41.3%)	119 (69.2%)	0 (0.0%)	131 (35.6%)	
Pathogen	3 (0.5%)	3 (1.7%)	0 (0.0%)	0 (0.0%)	
*Pharmacokinetic (PK) vs. pharmacodynamic (PD)*					
PK	190 (31.4%)	22 (12.8%)	40 (60.6%)	128 (34.8%)	**<0.001**
PD	416 (68.7%)	150 (87.2%)	26 (39.4%)	240 (65.2%)	
*Genetic variant vs. gene/protein expression*					
Genetic variant	484 (79.9%)	111 (64.5%)	66 (100.0%)	307 (83.4%)	**<0.001**
Gene/protein expression	122 (20.1%)	61 (35.5%)	0 (0.0%)	61 (16.6%)	
*Therapeutic area (TA)*					
Anesthetics	38 (6.3%)	14 (8.1%)	14 (21.2%)	10 (2.7%)	**<0.001**
Anti-infectives	19 (3.1%)	3 (1.7%)	1 (1.5%)	15 (4.1%)	
Antivirals	26 (4.3%)	4 (2.3%)	6 (9.1%)	16 (4.4%)	
Cardiovascular	43 (7.1%)	7 (4.1%)	6 (9.1%)	30 (8.2%)	
Hematology	79 (13.0%)	32 (18.6%)	4 (6.1%)	43 (11.7%)	
Neurology	33 (5.5%)	2 (1.2%)	5 (7.6%)	26 (7.1%)	
Oncology	217 (35.8%)	95 (55.2%)	3 (4.6%)	119 (32.3%)	
Other TAs	62 (10.2%)	2 (1.2%)	6 (9.1%)	54 (14.7%)	
Psychiatry	65 (10.7%)	10 (5.8%)	18 (27.3%)	37 (10.1%)	
Pulmonary	12 (2.0%)	2 (1.2%)	1 (1.5%)	9 (2.5%)	
Rheumatology	12 (2.0%)	1 (0.6%)	2 (3.0%)	9 (2.5%)	
*Recommendation category*					
Indication	194 (32.0%)	102 (59.3%)	0 (0.0%)	92 (25.0%)	**<0.001**
Contraindication	29 (4.8%)	0 (0.0%)	16 (24.2%)	13 (3.5%)	
Not recommended	82 (13.5%)	22 (12.8%)	39 (59.1%)	21 (5.7%)	
Dose adjustment	38 (6.3%)	2 (1.2%)	8 (12.1%)	28 (7.6%)	
Use with caution	65 (10.7%)	0 (0.0%)	0 (0.0%)	65 (17.7%)	
No dose adjustment	17 (2.8%)	2 (1.2%)	0 (0.0%)	15 (4.1%)	
Informational (none)	181 (29.9%)	44 (25.6%)	3 (4.6%)	134 (36.4%)	
Actionable	408 (67.3%)	126 (73.3%)	63 (95.5%)	219 (59.5%)	**<0.001**
Routine screening	206 (34.0%)	104 (60.5%)	n/a	102 (27.8%)	**<0.001**
*Quantitative characteristics*					
Years since publication	0.75 (0.19–1.77)	0.19 (0.19–0.81)	2.46 (0.58–4.04)	0.82 (0.42–1.77)	**<0.001**
Number of variants	0 (0–2)	1 (0–2)	11.5 (6–57)	0 (0–1)	**<0.001**

*CPG* clinical practice guideline, *CPIC* Clinical Pharmacogenetics Implementation Consortium, *FDA* U. S. Food & Drug Administration, *PGx* pharmacogenetics, Recs recommendations.

Data are presented as count (%) or median (interquartile range).

^*^P values are for the comparison of CPG vs. CPIC vs. FDA. Bolded *P* values indicate *P* < 0.05.

When comparing PGx recommendations by their source (CPG vs. CPIC vs. FDA), the FDA has published the most PGx recommendations (368), followed by CPGs (172) and CPIC (66). The PGx recommendations from those three sources significantly differed across all strata (Table 1; *P* < 0.001 for all comparisons). CPIC has only published PGx recommendations based on germline variants (100.0%), but most of the PGx recommendations published by CPGs are based on somatic (69.2%). Most PGx recommendations published by CPIC were based on the pharmacokinetics (60.6%), but most PGx recommendations published by CPGs and the FDA were based on pharmacodynamics (87.2% and 65.2%, respectively). Most PGx recommendations from all three sources were based on genetic variants, but CPGs had the most PGx recommendations based on gene/protein expression (35.5%). Oncology was the most common therapeutic area with PGx recommendations published by CPGs (55.2%) and the FDA (32.3%), but psychiatry is the therapeutic area with the most recommendations published by CPIC (27.3%). Most recommendations published by CPGs were categorized as Indication (59.3%), but most recommendations published by CPIC and FDA were categorized as Not Recommended (59.1%) and Informational/None (36.4%), respectively. CPIC has the highest percentage of PGx recommendations that were categorized as “actionable” (95.5%). CPGs recommended routine PGx screening more than recommendations from FDA (60.5% and 27.8%, respectively). CPGs had the most recently published recommendations (median (IQR) years since publication = 0.19 (0.19–0.81)). CPIC recommendations specifically mentioned the most variants (median (IQR) 11.5)(6–57)).

### Inconsistencies in the recommended biomarker/gene for each drug

Inconsistencies in the recommended biomarker/gene for each drug were summarized for all drugs and compared across several strata in Table 2. A total of 109 drugs were identified with at least two different PGx recommendations. Overall, 50.5% of drugs had inconsistent recommendations for the biomarker/gene associated with each drug. Drugs with both germline & somatic (*n* = 4) or germline & pathogen (*n* = 3) recommendations had the most biomarker inconsistencies (100% for both; *P* = 0.035 compared to drugs with only germline or somatic recommendations). Drugs with PGx recommendations for both genetic variant & gene/protein expression had significantly higher rates of biomarker inconsistencies (90%) than drugs with just genetic variant (44.4%) or gene/protein expression (55.6%) recommendations alone (*P* = 0.022). Rates of biomarker inconsistencies were similar among PK vs. PD recommendations and among therapeutic areas (*P* > 0.05).

**Table 2:** Inconsistencies in recommended biomarker/gene for each drug with at least two PGx recommendations.

Strata	Inconsistency in biomarker/gene	*P* value*
All drugs with at least two PGx Recs (*n* = 109)	55 (50.5%)	n/a
*Germline vs. somatic vs. pathogen*		
Germline (*n* = 47)	20 (42.6%)	**0.035**
Somatic (*n* = 55)	28 (50.9%)	
Germline & somatic (*n* = 4)	4 (100.0%)	
Germline & pathogen (*n* = 3)	3 (100.0%)	
*Pharmacokinetic (PK) vs. pharmacodynamic (PD)*		
PK (*n* = 25)	13 (52.0%)	0.327
PD (*n* = 81)	39 (48.2%)	
PK & PD (*n* = 3)	3 (100.0%)	
*Genetic variant vs. gene/protein expression*		
Genetic variant (*n* = 81)	36 (44.4%)	**0.022**
Gene/protein expression (*n* = 18)	10 (55.6%)	
Genetic variant & gene/protein expression (*n* = 10)	9 (90.0%)	
*Therapeutic area (TA)*		
Anesthetics (*n* = 7)	2 (28.6%)	0.719
Anti-Infectives (*n* = 3)	1 (33.3%)	
Antivirals (*n* = 6)	3 (50.0%)	
Cardiovascular (*n* = 2)	1 (50.0%)	
Hematology (*n* = 18)	9 (50.0%)	
Neurology (*n* = 4)	2 (50.0%)	
Oncology (*n* = 47)	25 (53.2%)	
Other TA (*n* = 4)	1 (25.0%)	
Psychiatry (*n* = 14)	10 (71.4%)	
Pulmonary (*n* = 2)	0 (0.0%)	
Rheumatology (*n* = 2)	1 (50.0%)	

*PGx* pharmacogenetics, *Recs* recommendations.

*P values are for the comparisons among each stratum. Bolded *P* values indicate *P* < 0.05.

### Composite of inconsistencies in the recommendations for each drug–gene pair

A composite of inconsistencies for each drug–gene pair was defined as any inconsistency in the following characteristics: recommendation category (e.g., contraindication vs. use with caution); recommendation group (e.g., poor vs. intermediate metabolizers); or routine screening (i.e., whether routine PGx screening is explicitly recommended). The composite of inconsistencies is summarized for all drug–gene pairs and compared across several strata in Table 3. A total of 158 drug–gene pairs were identified with at least two different PGx recommendations. Overall, 48.1% of drug–gene pairs had at least one inconsistency in their recommendations. The types of recommendations with the highest rates of the composite inconsistency are the following: germline (58.2%; *P* = 0.029), PK (93.9%; *P* < 0.001), genetic variants (53.1%; *P* = 0.046), and cardiovascular and psychiatry (both 100%; *P* < 0.001).

**Table 3:** Composite of inconsistencies in PGx recommendations for each drug–gene pair with at least two PGx recommendations.

Strata	Composite of inconsistencies	*P* value*
All drug–gene pairs with at least two PGx Recs (*n* = 158)	76 (48.1%)	n/a
*Germline vs. somatic vs. pathogen*		
Germline (*n* = 67)	39 (58.2%)	**0.029**
Somatic (*n* = 91)	37 (40.7%)	
*Pharmacokinetic (PK) vs pharmacodynamic (PD)*		
PK (*n* = 33)	31 (93.9%)	**<0.001**
PD (*n* = 125)	45 (36.0%)	
*Genetic variant vs. gene/protein expression*		
Genetic variant (*n* = 113)	60 (53.1%)	**0.046**
Gene/protein expression (*n* = 45)	16 (35.6%)	
*Therapeutic area (TA)*		
Anesthetics (*n* = 14)	1 (7.1%)	**<0.001**
Anti-Infectives (*n* = 3)	1 (33.3%)	
Antivirals (*n* = 3)	2 (66.7%)	
Cardiovascular (*n* = 5)	5 (100%)	
Hematology (*n* = 24)	12 (50%)	
Neurology (*n* = 6)	2 (33.3%)	
Oncology (*n* = 78)	33 (42.3%)	
Other TA (*n* = 5)	3 (60%)	
Psychiatry (*n* = 16)	16 (100.0%)	
Pulmonary (*n* = 2)	1 (50%)	
Rheumatology (*n* = 2)	0 (0.0%)	

*PGx* pharmacogenetics, *Recs* recommendations.

*P values are for the comparisons among each stratum. Bolded *P* values indicate *P* < 0.05.

### Specific types of inconsistencies in the recommendations for each drug–gene pair

The specific types of inconsistencies, that composed the composite inconsistency described above, were summarized for all drug–gene pairs with at least two different recommendations (*n* = 158), and they were stratified by germline vs. somatic in Table 4. Overall, the most common type of inconsistency among drug–gene pairs was the recommendation group (35.4%). Germline recommendations had significantly more inconsistencies in the recommendation category than somatic (49.3% vs. 15.4%; *P* < 0.001), “actionable” recommendations (28.4% vs. 15.4%; *P* = 0.047), and recommendation group (47.8% vs. 26.4%; *P* = 0.006). However, somatic recommendations had significantly more inconsistencies in routine screening recommendations than germline (25.3% vs. 1.5%; *P* < 0.001).

**Table 4:** Specific characteristics & inconsistencies in PGx recommendations by drug–gene pair and germline vs. somatic.

	All (*n* = 158)	Germline (*n* = 67)	Somatic (*n* = 91)	*P* value*
*Specific characteristics of recommendations*				
Recommendation category				
Indication	83 (52.5%)	5 (7.5%)	78 (85.7%)	**<0.001**
Contraindication	1 (0.6%)	1 (1.5%)	0 (0.0%)	
Not recommended	39 (24.7%)	37 (55.2%)	2 (2.2%)	
Dose Adjustment	5 (3.2%)	5 (7.5%)	0 (0.0%)	
Use with caution	0 (0.0%)	0 (0.0%)	0 (0.0%)	
No dose adjustment	2 (1.3%)	2 (3.0%)	0 (0.0%)	
Informational (none)	28 (17.7%)	17 (25.4%)	11 (12.1%)	
Actionable recommendations	128 (81.0%)	48 (71.6%)	80 (87.9%)	**0.010**
Routine screening	84 (53.2%)	10 (14.9%)	74 (81.3%)	**<0.001**
Years since publication	0.19 (0.19–1.23)	1.51 (1.23–5.43)	0.19 (0.07–0.19)	**<0.001**
Number of variants	1 (0–6)	2 (0–48)	1 (1–2)	**0.036**
*Specific inconsistencies among recommendations*				
Inconsistencies in recommendation category	47 (29.8%)	33 (49.3%)	14 (15.4%)	**<0.001**
Inconsistencies in actionable recommendations	33 (20.9%)	19 (28.4%)	14 (15.4%)	**0.047**
Inconsistencies in routine screening	24 (15.2%)	1 (1.5%)	23 (25.3%)	**<0.001**
Inconsistencies in recommendation group	56 (35.4%)	32 (47.8%)	24 (26.4%)	**0.006**

*PGx* pharmacogenetics.

Data are presented as count (%) or median (interquartile range).

*P values are for the comparison of germline vs. somatic. Bolded *P* values indicate *P* < 0.05.

A total of 15 drug–gene pairs had PGx recommendations from all three sources (CPG, CPIC, and FDA). Fourteen of those drug–gene pairs (93.3%) had at least one type of inconsistency in their PGx recommendations. The specific types of inconsistencies identified for those 15 drug–gene pairs are displayed in Table 5. Abacavir/*HLA-B* is the only drug–gene pair for which all three recommendations from the CPG, CPIC, and FDA are consistent for all aspects of the recommendation. Azathioprine/*TPMT* is the single drug–gene pair for which there is an inconsistency in all aspects of the recommendations: the biomarker/gene, recommendation category, clinically “actionable”, recommendation group, and routine screening.

**Table 5:** Specific inconsistencies in PGx recommendations for drug–gene pairs with recommendations from all three sources: CPIC, FDA, and professional CPGs.

Drug–gene pair	Specific types of inconsistencies present				
	Biomarker/gene	Recommendation category	Clinically actionable	Recommendation group	Routine screen
Abacavir/*HLA-B*					
Azathioprine/*TPMT*	X	X	X	X	X
Capecitabine/*DPYD*	X	X	X	X	
Citalopram/*CYP2C19*	X	X	X	X	
Clopidogrel/*CYP2C19*		X	X	X	
Fluorouracil/*DPYD*		X	X	X	
Fluvoxamine/*CYP2D6*	X	X	X	X	
Ivacaftor/*CFTR*		X		X	
Mercaptopurine/*TPMT*	X	X		X	
Paroxetine/*CYP2D6*	X	X	X	X	
Sevoflurane/*RYR1*	X	X		X	
Tamoxifen/*CYP2D6*	X	X	X	X	
Voriconazole/*CYP2C19*		X	X	X	
Warfarin/*CYP2C9*	X	X	X	X	
Warfarin/*VKORC1*	X	X	X	X	

*CPG* clinical practice guidelines for professional medical organizations, *CPIC* Clinical Pharmacogenetics Implementation Consortium, *FDA* U.S. Food and Drug Administration.

Marked boxes indicate the presence of the respective.

## Discussion

While there are many different challenges facing the effective and routine clinical implementation of PGx^7,8,10^, inconsistencies in clinical PGx recommendations may pose an additional barrier. The specific biomarkers/genes recommended for half of the drugs were inconsistent. Inconsistencies in the biomarker or gene are important because it is unclear to the clinician which genetic tests should be ordered for the drug that is being prescribed. For example, it is unclear to prescribers of azathioprine whether only a *TPMT* test should be ordered^13^, or a *NUDT15* test as well^14^. Similarly, approximately one-half of all drug–gene pairs with at least two different recommendations had at least one type of inconsistency in the PGx recommendation. Of the specific types of inconsistencies assessed, inconsistencies were most commonly related to the specific patient groups subject to the PGx recommendations. Inconsistencies in the patient groups are important because it is unclear which patients should have changes made to their therapy based on their genotype-predicted phenotype. For example, it is unclear whether clopidogrel should be avoided in patients that are *CYP2C19* intermediate metabolizers. CPIC recommends alternative therapy for both *CYP2C19* poor and intermediate metabolizers^15^, whereas the FDA only recommends alternative therapy in *CYP2C19* poor metabolizers^16^. We also found that almost one-third of drug–gene pairs had inconsistencies among the recommendation category (e.g., contraindication vs. use with caution). These discrepancies are important because it is unclear to the clinician whether the drug is truly contraindicated, or it can still be used with caution. For example, CPIC recommends to avoid amitriptyline use in *CYP2D6* poor metabolizers^17^, but the FDA only discusses amitriptyline use in *CYP2D6* poor metabolizers as a warning/precaution^18^. Approximately one-in-seven drug–gene pairs were inconsistent according to whether or not routine genetic screening was recommended. These types of inconsistencies are important because it is unclear to clinicians whether genetic testing should be performed in all patients treated with a certain drug, or only certain patients. An example of an inconsistency in routine genetic testing would be for azathioprine. The CPG states “*TPMT* genotyping is recommended as a useful adjunct to a regimen for prescribing azathioprine^13^,” but the FDA only recommends evaluating TPMT deficiency in patients that suffer from severe myelosuppression^14^. Our results also show inconsistencies in the “clinical actionability” of approximately one-fifth of PGx recommendations. These types of inconsistencies are especially confusing to clinicians because it is unclear whether the action is warranted or not. For example, CPIC recommends to avoid the use of 5-fluorouracil in patients that are DPYD poor metabolizers^19^, but the NCCN does not support the use of pre-emptive DPYD genotyping, despite the increased risk of toxicity^20^. Overall, these results provide evidence of prevalent inconsistencies among clinical PGx recommendations from the most prominent U.S. guidance sources. If the most prominent sources of PGx information in the U.S. cannot agree on PGx recommendations, then that may erode the public’s perception of PGx.

A previous investigation by Filipski et al. characterized PGx recommendations from FDA drug labels and U.S. professional medical and technology assessor organizations^8^. However, they restricted their analysis to only drug-metabolizing enzymes. Their search was performed through August 2015, and they found 189 biomarker–drug pairs, of which only 84 met their inclusion criteria. Within that smaller subset of PGx recommendations, they only compared two recommendation elements: the therapeutic recommendation (e.g., avoid use vs. use with caution) and the strength of the recommendation (e.g., recommended vs. essential). They did not specifically compare other recommendation elements that are important in clinical care, such as the patient group subject to the recommendation or the specific genetic variants recommended. Their evaluation of the FDA labels was quantitative, but when they compared the FDA labels to independent technology assessors, they only summarized the numbers of recommendations that were also actionable. Therefore, our study builds upon their work by providing a more comprehensive search and evaluation of all currently published clinical PGx recommendations and multiple elements of the recommendation.

Bank et al. published a comparison of the guidelines from CPIC and the Dutch Pharmacogenetics Working Group (DPWG)^9^. That study compared several recommendation elements, such as differences in terminology, allele classification, genotype-to-phenotype conversion, numbers of variants, and therapeutic recommendations. They found differences in therapeutic recommendations for 16 out of the 27 drug–gene pairs evaluated (59.3%), which is similar to the rate of composite inconsistencies identified in this study (48.1%). The study by Bank et al. provides important insight into the processes for developing PGx clinical recommendations. This study builds upon the work by Bank et al. by providing more clinically relevant information for clinicians in the U.S because clinicians in the U.S. typically get clinical PGx recommendations from either CPIC, FDA, or CPGs, as opposed to only the DPWG vs. CPIC. A previously published perspective paper by Luzum et al. evaluated three drug–gene pairs^10^. That paper performed an in-depth comparison of the levels of evidence for those three gene–drug pairs, but it did not provide a comprehensive or quantitative analysis of inconsistencies overall.

Shekhani et al. performed a thorough investigation of discrepancies among clinical PGx recommendations from the FDA, CPIC, DPWG, and European regulatory agencies (e.g., the European Medicines Agency (EMA))^21^. Shekhani et al. found a consensus rate of 18% among all agencies regarding clinical actionability, which is similar to our consensus rate of 20.9% for clinical actionability. A critical piece that is missing from the study by Shekhani et al. is that they did not evaluate PGx recommendations from CPGs. In our clinical experience, CPGs are the primary source of clinical recommendations for many clinicians in the U.S., even when it comes to PGx recommendations. Programs that advocate for the adoption of CPGs by clinicians, such as the AHA’s “Get with the Guidelines” program^22^, support this point. Moreover, clopidogrel PGx is an example in which a CPG was written specifically in response to a black box warning by the FDA^23^. Therefore, we believe that our study is more clinically relevant than the study by Shekhani et al., at least for clinicians in the U.S., as our study included a critical source of clinical recommendations (CPGs).

The high rate of inconsistencies among clinical PGx recommendations likely stems from multiple causes. The level of scientific evidence required to inform “clinically actionable” PGx remains a controversial issue. There are even inconsistencies in the manner in which different organizations appraise and grade scientific evidence^24–26^. CPIC defines a high level of evidence as “…consistent results from well-designed, well-conducted studies.”^24^ This definition does neither specifically require the randomized controlled trial (RCT) study design nor do guidelines from the NCCN (e.g., *TPMT* genotyping for thiopurine dosing)^27^. Conversely, it seems that AHA/ACC guidelines apply the same evidence-grading criteria to PGx recommendations as they do for other therapeutic recommendations, with the highest level of evidence requiring at least one RCT plus corroboration by at least one more RCT or high-quality registry^28^. To our knowledge, the FDA has not defined the level of evidence required for PGx information to be incorporated into drug labels^29^. Most PGx information incorporated into FDA drug labels is based on dedicated studies performed by the drug sponsor during approval, with PGx labeling updates limited to situations in which a new major safety issue is identified post-approval^30^. The single drug–gene pair in which all three sources agree on all elements of the recommendation, abacavir/*HLA-B*, likely occurs because of the high level of evidence supporting the drug–gene pair. The large, double-blind, prospective, and randomized PREDICT-1 clinical trial provides unequivocal evidence for the benefit of PGx testing for preventing adverse events from abacavir^31^.

Differences in the organizational missions of the various guidance sources are also likely to explain the inconsistencies in clinical PGx recommendations. This is evidenced by the fact that CPIC guidelines only provide recommendations for how available genetic results should be used to inform optimal drug therapy^32^. In contrast, the purpose of FDA drug labeling is to summarize the most important scientific information needed to ensure safe and effective drug use, which may or may not include recommendations about whether a genetic screen should be ordered (21 CFR 201.57). We recognize that the major purpose of the FDA drug label is only to provide information and not recommendations. Therefore in this study, the specific language of the FDA labels was analyzed. If the language in the FDA label was not simply informational, but provided recommendations, then it was categorized as a recommendation because clinicians can interpret it that way. It may be unreasonable to think that FDA labeling can/should have the same recommendations as CPGs because the overall purpose of CPGs is different from the purpose of CPIC and FDA publications. Each professional organization (e.g., AHA, NCCN) has its own unique mission. CPGs evaluate the available evidence, translate that evidence into recommendations, and thereby inform clinical and policy decision-making^25^. A factor that was particularly unique for CPIC was that CPIC recommendations included many more specific genetic variants (~tenfold more) than CPGs or FDA labels. Including more genetic variants may be helpful for clinicians when deciding which genetic tests to order, and it may more accurately predict the phenotype in the patient (e.g., normal vs. intermediate metabolizer).

How can these inconsistencies become more aligned? We propose three potential solutions. First, stronger evidence supporting PGx (i.e., RCTs demonstrating clinical utility) may help to improve consistency among clinical recommendations, as was the case with abacavir/*HLA-B* described above. However, given that we identified >600 clinical PGx recommendations already in the literature, it is impractical to perform that many RCTs. Additionally, there exist a number of other considerations specific to PGx (e.g., the difficulty of recruiting patients with variants of low minor allele frequency, ethical concerns related to exposing patients at high risk of toxicity to a drug) that limit the feasibility of PGx RCTs, as many have identified^10,33–36^. Another potential solution could be formal engagement between PGx expert consortiums and professional organizations, such as with expert panels. Indeed, the 2017 guideline on high blood pressure in adults included ten other organizations as co-authors along with the AHA^37^. Therefore, it seems likely that a similar collaboration including CPIC could be accomplished as well. A third potential solution could be for organizations to endorse a single source for PGx clinical recommendations. For example, the American Society for Clinical Pharmacology and Therapeutics and the American Society of Health-System Pharmacists endorse CPIC guidelines^38^. The NCCN guidelines already cite CPIC for their PGx recommendations^39^. If other professional organizations would endorse CPIC as well, then that would resolve some more inconsistencies.

Why is it important to align PGx recommendations? These inconsistencies create different problems for different stakeholders: clinicians, health systems, third-party payers, and patients. From the clinician’s perspective, it is unclear how to manage a patient if three different organizations recommend three different courses of action. We demonstrate this issue with a two-part patient case in the Supplementary Figs. 1 and 2. For health systems choosing to implement PGx, it is difficult to decide which PGx recommendations to follow. For example, which PGx tests should be implemented across the health system? The FDA provides PGx information for 368 drug–gene pairs, whereas CPIC only provides information for 66. Inconsistent PGx recommendations prevent straightforward implementation of clinical decision support within electronic medical records. For example, if a patient is being prescribed clopidogrel, should a “Best Practice Alert” fire for only patients that are *CYP2C19* poor metabolizers (as recommended by the FDA)^16^, or should it also fire for *CYP2C19* intermediate metabolizers (as recommended by CPIC)^15^? Evidence shows that health systems are not necessarily choosing to follow a single source for PGx recommendations, but rather they are tailoring their PGx implementation programs based on a combination of recommendations from both CPIC and the FDA^1^. (That study did not investigate the influence of CPGs in PGx clinical implementation)^1^. However, if there was a single source of PGx information to follow, then health systems could avoid having to “reinvent the wheel” for each new PGx implementation program. Inconsistent PGx recommendations may also affect reimbursement for PGx tests by third-party payers. For example, United Health Care covers multigene panels to guide therapy decisions for antidepressants and antipsychotics, but not for any other indication, including but not limited to pain management, cardiovascular drugs, anthracyclines, or polypharmacy^40^. The company not only cites the primary literature supporting those coverage decisions, but they also cite recommendations from CPIC, FDA, and CPGs in their coverage decisions. Finally, patients may be confused by inconsistent PGx recommendations. Millions of patients will soon have access to their PGx test results through 23andMe^11^. It is currently unknown where patients will go for PGx information, but the FDA recommends discussing their 23andMe PGx results with their healthcare provider. FDA drug labels include a patient counseling information section for providers (section 17), but only 21% of FDA drug labels with PGx information also include the PGx information in the patient-targeted sections^41^.

While inconsistencies in clinical PGx recommendations may pose a significant barrier hindering the clinical implementation of PGx, PGx implementation efforts are already demonstrating that they can overcome this hurdle. For instance, clopidogrel/*CYP2C19*, which has inconsistencies in three of five recommendation elements, was the only drug–gene pair for which PGx testing was implemented at all seven health systems within the Translational Pharmacogenetics Program of the NIH Pharmacogenomics Research Network^1^. This example highlights the ability of large health systems to successfully navigate this barrier, but clinicians practicing outside of medical centers with established PGx initiatives would seemingly be less likely to pursue PGx testing in the face of recommendation inconsistencies.

Our study has several limitations. We were unable to directly assess the evidentiary basis for all of the clinical PGx recommendations among all guidance sources. Initial drug-labeling decisions are often based on proprietary data submitted to the FDA directly by drug developers. Deciphering whether inconsistencies in recommendations are based on distinct interpretations of the same evidence, or on different levels of evidence, is a key consideration to inform potential strategies to harmonize clinical PGx recommendations. Another limitation of our work is that we only assessed major U.S. guidance sources (with the exception of the European Malignant Hyperthermia Guidelines). Our data collection included EGAPP, which is no longer actively updating their PGx recommendations, but omitting EGAPP recommendations only slightly changed the results and does not affect the overall conclusions. By omitting EGAPP, the overall rate of composite inconsistencies would change from 48.1 to 45.7%, and the overall rate of inconsistencies in biomarkers would change from 50.5 to 47.7%. The prevalence of clinical PGx recommendation inconsistencies is likely different in other regions of the world (e.g., Europe) where there are different PGx organizations (e.g., the Dutch Pharmacogenomics Working Group), drug regulatory bodies (e.g., European Medicines Agency), and professional medical organizations (e.g., the European Society of Cardiology). However, we believe that this work can serve as an example for future, similar analyses in other regions of the world. Our search strategy prioritized CPIC and FDA, and then we searched CPGs only for those drugs covered by CPIC or FDA PGx recommendations. Therefore it is possible that PGx recommendations in CPGs, which are not in CPIC or FDA PGx recommendations, would have been missed. Our search strategy would also have missed the possibility of two different CPGs with inconsistent PGx recommendations. The FDA recently released a new “Table of Pharmacogenetic Associations” on February 25, 2020^42^. We chose to keep our data collection from the FDA Table of Pharmacogenomic Biomarkers in Drug Labeling instead, because when the FDA released the new table, the FDA stated “information provided in this (new) table is limited to certain pharmacogenetic associations only and does not provide comprehensive information needed for safe and effective use of a drug…healthcare providers should refer to FDA-approved labeling for prescribing information.” Regardless, our analyses still included ~88% of drug–gene pair recommendations from the new FDA tables. In addition, PGx recommendations from the new FDA table that are not included in our analyses largely examine recently approved medications that are not yet mentioned in CPIC guidelines or professional CPGs. Therefore, those new drug–gene pairs would not affect our inconsistency analysis, which requires at least two different published PGx recommendations.

In conclusion, our work provides comprehensive and quantitative evidence to demonstrate the prevalence of inconsistencies in clinical PGx recommendations from major U.S. sources. Given these prevalent inconsistencies, the question remains: which PGx recommendations should clinicians follow? Future directions of this work should focus on understanding the underlying factors associated with these inconsistencies, as well as exploring the influence of these inconsistencies on clinical PGx implementation programs and clinical outcomes.

## Methods

### Data collection

First, we collected the most current clinical PGx recommendations via manual screening of CPIC guidelines and FDA drug labels. Then we searched for PGx recommendations for the same drugs in CPGs from professional medical organizations and independent technology assessors in the U.S. All data are provided in the Online Supplementary Information. Data sources were reviewed for updated recommendations through May 24, 2019. Clinical PGx recommendations that mentioned a specific gene or gene product to be considered during the administration of an FDA-approved drug entity was considered a “drug–gene pair.” Collected data included (1) the specific therapeutic management (e.g., dose adjustment); (2) the patient groups subject to the recommendation (e.g., CYP2D6 poor vs. intermediate metabolizers); (3) the biomarker involved (e.g., the specific gene or protein expression); (4) the specific genetic variants included in the recommendation (when available); (5) whether routine screening is explicitly recommended; (6) the placement of the recommendation if in an FDA drug label (e.g., the “Warnings & Precautions” section); and (7) the date the recommendations were published (defined as when the recommendation was first available online or in print). The number of variants within each drug–gene pair recommendation was calculated based on all unique variants or variant alleles included within each guidance document, including those listed within online supplementary information. The time since the publication was calculated as the years elapsed between the publication date of the guidance source document and the final date of data collection (May 24, 2019). For FDA drug labels, it was calculated from the most recent version of the drug label in which the PGx information was contained. The therapeutic area was assigned based on the FDA Division that approved the product, as shown in the approval letter in the Drugs@FDA database.

CPIC guidelines were accessed via the “Genes-Drugs” section of the CPIC website: https://cpicpgx.org/genes-drugs/. FDA drug labels were accessed via the Drugs@FDA database (https://www.accessdata.fda.gov/scripts/cder/drugsatfda/) for drugs identified in the current list (updated on March 26, 2019) of the FDA’s Table of Pharmacogenomic Biomarkers in Drug Labeling^43^. CPGs from professional organizations or independent technology assessors for drug–gene pairs identified in CPIC guidelines or FDA drug labels were found via (1) search of guidelines.gov, PubMed, and Google Scholar with keywords that included the specific drug and biomarker; (2) review of the references cited in the most recent version of CPIC guidelines, when available; (3) review of guidelines of U.S. professional organizations in the therapeutic area of the drug in the drug–gene pair; and (4) clinician consultation. CPGs specific to individual hospitals or health systems (e.g., recommendations from the Veterans Health Administration Clinical Pharmacogenetics Subcommittee^44^) were not included. One exception to using U.S. based CPGs was the CPGs from the European Malignant Hyperthermia Group. That exception was based on the absence of a U.S.-based CPG, and clinician consultation stating that U.S. anesthesiologists generally follow the European CPG (personal communication). When more than one CPG included recommendations for a drug–gene pair, then only a single CPG was selected for analysis. We prioritized the selection of the CPG based on the following criteria: first, the CPG with PGx recommendations for the broadest range of therapeutic indications was selected. The decision to select a CPG with a broad range of indications (e.g., National Academy of Clinical Biochemistry) versus a CPG for a specific indication (e.g., American Gastroenterological Association) was made to maximize consistency in our comparisons. CPIC and FDA typically make PGx recommendations based on broad indications. Next (if applicable), the CPG with the most recent date of publication was selected. If a CPG for a broad range of indications was not available, then the most recently published, indication-specific CPG was selected. If included in a CPIC guideline or FDA label, then “emerging biomarkers” in NCCN guidelines were collected as well.

### Categorization of clinical PGx recommendations

Clinical PGx recommendations were categorized as the following: presence or absence of the biomarker required for drug Indication; Contraindication in the presence of the biomarker; administration Not Recommended in the presence of the biomarker; Dose Adjustment recommended in the presence of the biomarker; Use With Caution in the presence of the biomarker; No Dose Adjustment recommended in the presence of the biomarker; and Informational (None) when information related to the drug–gene interaction was provided but no explicit therapeutic management recommendation was given. In the case of FDA drug labels, the location of information describing the management of the drug–gene pair was also used to assign a recommendation category when specific label language was not available. Recommendations that contained non-compulsory language (e.g., “consider using an alternative agent”) were categorized as the suggested recommendation but noted to be non-compulsory. For consistency in our comparisons, only a single therapeutic approach per PGx recommendation could be analyzed. Therefore, when more than one therapeutic approach was provided in a PGx recommendation (e.g., avoid a drug or 50% dose reduction), then the strongest recommendation was selected for our analysis. We selected the strongest recommendation based on the following hierarchy: Contraindication > Not Recommended > Dose Adjustment > Use with Caution > No Dose Adjustment.

Within our analyses, Indication, Contraindication, Not Recommended, Dose Adjustment, and Use With Caution were defined as “clinically actionable”, while recommendations of No Dose Adjustment and Informational (None) were not. Use With Caution was defined as clinically actionable, as consistent with a recent FDA perspective^45^, because it is expected that additional clinical monitoring would be utilized for patients meeting this recommendation (e.g., more frequent labs drawn or more frequent visits scheduled with providers).

### Categorization of the types of inconsistencies in clinical PGx recommendations

Inconsistencies in clinical PGx recommendations were defined as situations in which a drug–gene pair had contradictory or distinct recommendations from two or more sources. The first element of the recommendation that was analyzed for each of the drugs was the specific biomarker contained within each drug–gene pair. Inconsistencies in biomarkers were analyzed separately; since comparison of the other recommendation elements (described next) could only be performed for drug–gene pairs with a consistent biomarker from multiple sources. Then the following types of inconsistencies were analyzed separately: (1) recommendation category (e.g., one source states that the drug–gene pair is a contraindication, whereas another source only recommends using with caution); (2) defined as “clinically actionable”; (3) patient groups subject to the recommendation, including groups based on individual variants, haplotypes, phenotypes, enzyme activity, or enhanced gene expression (e.g., one source recommends to adjust the dose in both CYP2C19 intermediate and poor metabolizers, whereas another source only recommends to adjust the dose in poor CYP2C19 metabolizers); (4) whether routine screening was explicitly recommended or not. Recommendation categories were still considered inconsistent if a drug–gene pair had one recommendation that was only categorized as “Informational/None” or “No Dose Adjustment” and another recommendation as something actionable. The absence of a recommendation from one source but the presence of a recommendation in another source was not counted as an inconsistency.

Recommendations categorized as Contraindication and Not Recommended were considered consistent. A composite measure of recommendation inconsistencies was categorized as the presence of one or more inconsistencies within the (1) recommendation category, (2) the patient group, and/or (3) whether routine screening was recommended. In other words, inconsistency was a difference within an individual recommendation element described above, whereas the composite was the presence of any type of inconsistency. Inconsistencies in whether routine screening was recommended were only analyzed between FDA drug labels and professional CPGs, since CPIC recommendations do not address whether genetic tests should be ordered^32^.

### Statistical analysis

Continuous variables were described by the median (IQR) and compared by Wilcoxon rank-sum or Kruskal–Wallis tests. Categorical variables were described by counts and percentages and compared by chi-square or Fisher’s exact test when necessary. Data were summarized overall and compared by the following strata: CPG vs. CPIC vs. FDA, germline vs. somatic, PK vs. PD, genetic variant vs. gene/protein expression, and therapeutic area. Comparisons were made with distinct samples and not with repeated measures. All statistical analyses were performed using SAS v9.4, and the level of statistical significance was defined as *P* < 0.05 (two-sided).

### Reporting summary

Further information on research design is available in the Nature Research Reporting Summary linked to this article.

## Supplementary information

Supplementary Information

Supplementary Data

Reporting Summary Checklist

## Data Availability

All data are available in the Supplementary File. Table 1 analyzed all 606 PGx recommendations collected, and the corresponding tab in the online supplementary excel file has been named “AllPGxRecs(Table 1)”. Table 2 analyzed every drug with >1 PGx recommendation, and the corresponding tab in the Supplementary excel file has been named “DrugsWith>1Rec(Table 2)”. Tables 3 and 4 analyzed every drug–gene pair with >1 PGx recommendation, and the corresponding tab in the Supplementary excel file has been named “DrugGenePairs >1Rec (Tables 3 & 4)”. Table 5 shows the drug–gene pairs with three different PGx recommendations, and the corresponding tab in the online supplementary Excel file has been named “DrugGenePairsWith3Recs(Table 5)”.
