# Supplementary material for: Prevalence and types of inconsistencies in clinical pharmacogenetic recommendations among major U.S. sources
Source: NPJ Genom Med. 2020 Oct 30;5:48. doi: 10.1038/s41525-020-00156-7 (PMC7603298; doi:10.1038/s41525-020-00156-7)
Supplement: Supplementary file 1 — Supplementary Information [file 41525_2020_156_MOESM1_ESM.pdf]

SUPPLEMENTARY INFORMATION

SUPPLEMENTARY DATA SET

All of the data used in the analysis is provided in the Excel dataset provided online. Table 1 in the manuscript analyzed all 606 PGx recommendations collected, and the corresponding tab in the online supplementary Excel file has been named “AllPGxRecs(Table1)”. Table 2 in the manuscript analyzed every drug with >1 PGx recommendation, and the corresponding tab in the online supplementary Excel file has been named “DrugsWith>1Rec(Table2)”. Tables 3 and 4 in the manuscript analyzed every drug-gene pair with >1 PGx recommendation, and the corresponding tab in the online supplementary Excel file has been named “DrugGenePairs>1Rec(Tables3&4)”. Table 5 in the manuscript shows the drug-gene pairs with 3 different PGx recommendations, and the corresponding tab in the online supplementary Excel file has been named “DrugGenePairsWith3Recs(Table5)”.

SUPPLEMENTARY FIGURES

Supplementary Figure 1. Part 1 of a patient case demonstrating how inconsistencies in clinical PGx recommendations from the 3 major U.S. sources lead to unclear patient management. AA = African-American; AHA = American Heart Association; CC = chief complaint; CPIC = Clinical Pharmacogenetics Implementation Consortium; CYP2C19 = cytochrome P450 family 2 subfamily C member 19; DES = drug eluting stent; ED = emergency department; FDA = U.S. Food & Drug Administration; HL = hyperlipidemia; HPI = history of present illness; HTN = hypertension; LAD = left anterior descending; meds = medications; PGx = pharmacogenetics; PMH = past medical history; qd = every day; qhs = at bedtime; STEMI = ST segment elevated myocardial infarction

CC: C.R. is a 62 yo AA male that presents to your clinic today for hospital discharge f/u.
HPI: Three weeks ago C.R. presented to the ED with STEMI. He was loaded with 600mg clopidogrel and sent emergently to the cath lab. DES implantation in the LAD was successful.
PMH: HTN, HL, depression
Meds: aspirin 81mg qd, clopidogrel 75mg qd, atorvastatin 40mg qd, metoprolol XL 100mg qd, amitriptyline 75mg qhs, lisinopril 10mg qd

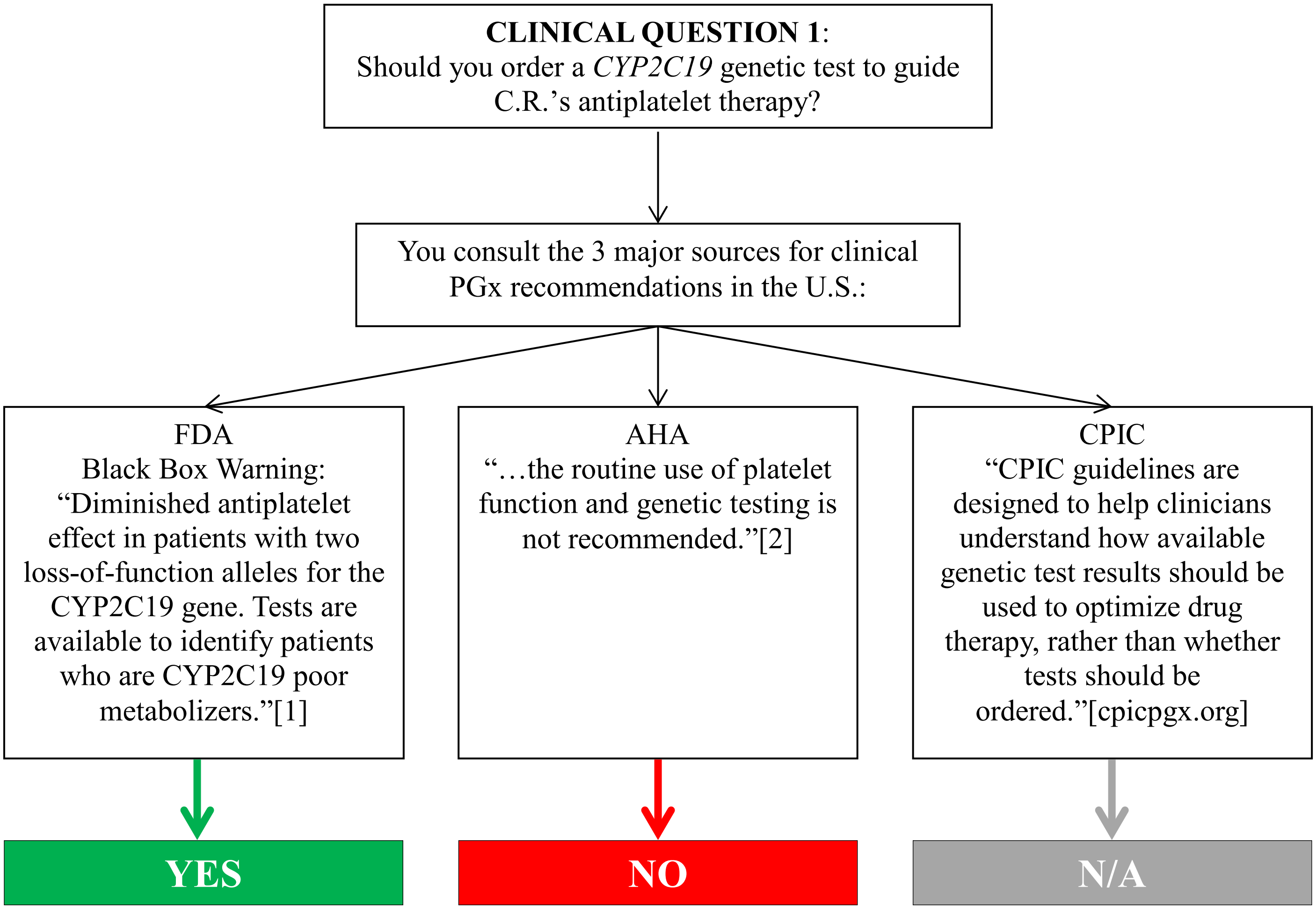

Supplementary Figure 2. Part 2 of a patient case demonstrating how inconsistencies in clinical PGx recommendations from the 3 major U.S. sources lead to unclear patient management. AHA = American Heart Association; CPIC = Clinical Pharmacogenetics Implementation Consortium; CYP2C19 = cytochrome P450 family 2 subfamily C member 19; FDA = U.S. Food & Drug Administration; PGx = pharmacogenetics

UPDATE:

Later that day you find a pdf in the "Media" tab of C.R.'s chart. It is the report from a multi-gene panel, which was ordered by C.R.'s psychiatrist to guide C.R.'s antidepressant therapy. (Multi-gene panels for guiding antidepressant therapy are covered by C.R.'s United Health Care insurance plan). It indicates that C.R. is a CYP2C19 Intermediate Metabolizer.

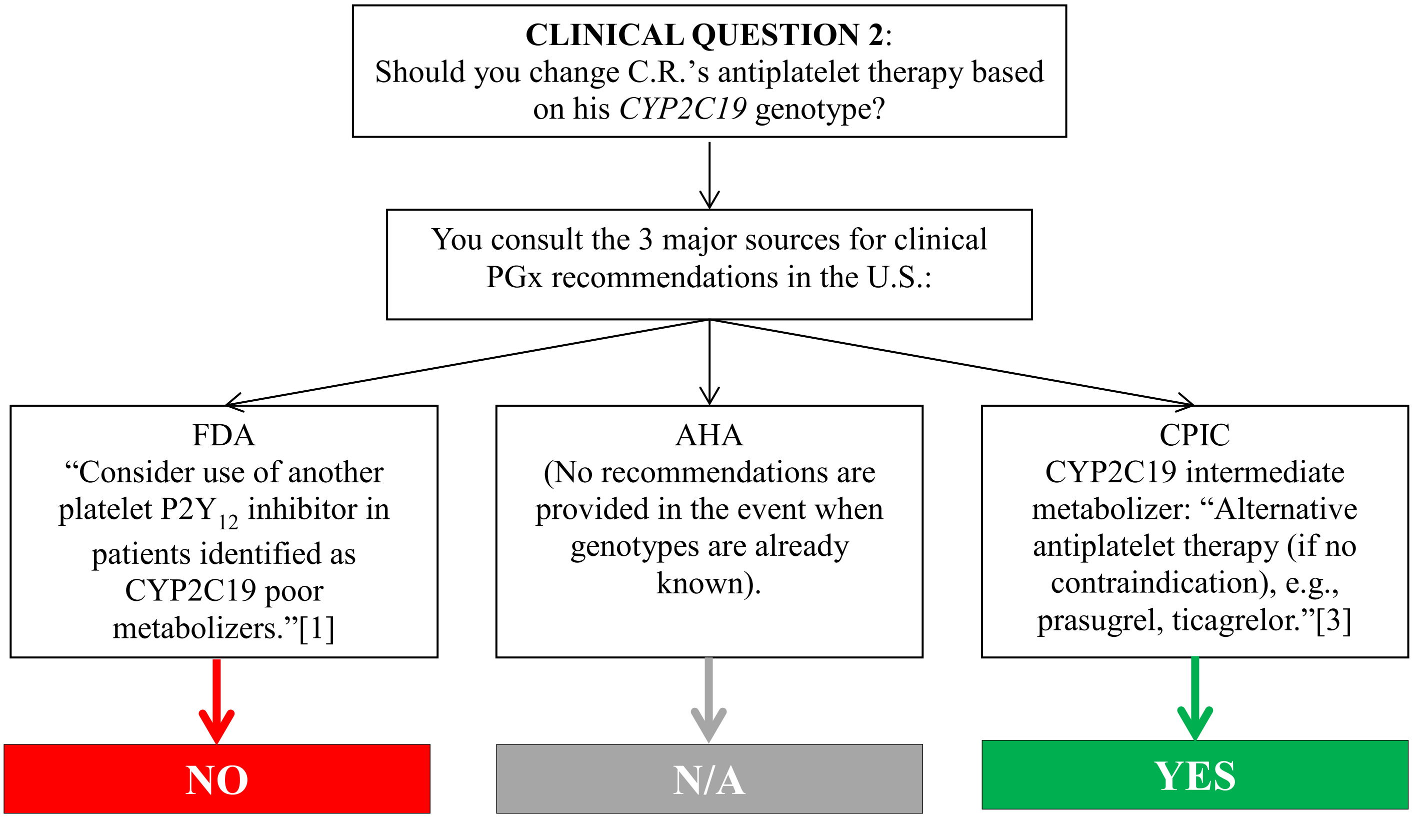

REFERENCES

1. U.S. Food & Drug Administration. Clopidogrel Prescribing Information. URL: https://www.accessdata.fda.gov/drugsatfda_docs/label/2019/020839s072lbl.pdf (2019).

2. Levine GN, Bates ER, Bittl JA et al. 2016 ACC/AHA Guideline focused update on duration of dual antiplatelet therapy in patients with coronary artery disease: a report of the American College of Cardiology/American Heart Association Task Force on Clinical Practice Guidelines. *J. Am. Coll. Cardiol*. 68(10), 1082–1115 (2016).

3. Scott, S. A. *et al.* Clinical Pharmacogenetics Implementation Consortium guidelines for CYP2C19 genotype and clopidogrel therapy: 2013 update. *Clinical pharmacology and therapeutics* **94**, 317-323, (2013).
